# Body composition reference charts for UK infants and children aged 6 weeks to 5 years based on measurement of total body water by isotope dilution

**DOI:** 10.1038/s41430-019-0409-x

**Published:** 2019-02-26

**Authors:** Jonathan C. K. Wells, Peter S. W. Davies, Mary S. Fewtrell, Tim J. Cole

**Affiliations:** 1 Childhood Nutrition Research Centre, UCL Great Ormond Street Institute of Child Health, London, UK; 2 Population, Policy, and Practice Programme, UCL Great Ormond Street Institute of Child Health, London, UK; 3 Child Health Research Center, Centre for Children’s Health Research, University of Queensland, Brisbane, QLD Australia

**Keywords:** Biomarkers, Developmental biology

## Abstract

**Background:**

Until recently, pediatric body composition reference data were very limited, hindering interpretation of measurements. In the last decade, such data emerged for several techniques for children ≥ 5 years, but equivalent data for younger age groups remain lacking, due to their poor compliance with most techniques.

**Objectives:**

To provide reference data for use in clinical practice and research from 6 weeks to 5 years, that are based on measurements of total body water (TBW) by isotope dilution.

**Design:**

The data on anthropometry and TBW were available from studies of 463 infants and children aged 6 weeks to 7 years, conducted between 1988 and 2010. Both breast-fed and formula-fed infants were included. TBW was measured by ^2^H- or ^18^O-labeled water, and converted to fat-free mass (FFM) using published hydration coefficients. Reference charts and SD scores (SDS) were constructed for FFM, fat mass (FM), FFM index and FM index for each sex, using the lambda-mu-sigma method.

**Results:**

Both sexes were significantly heavier and longer than UK 1990 reference data (*p* < 0.01), but did not differ in body mass index SDS. Breast-fed infants were longer than formula-fed infants but did not differ in body composition.

**Conclusions:**

These reference data will enhance the ability of clinicians to assess and monitor body composition and FFM/FM accretion in clinical practice in younger age groups. Total body water can be measured in most patients, though abnormalities of hydration must be addressed. However, the centiles do not overlap exactly with those published for older age groups, limiting comparability between younger and older children.

## Introduction

Growth charts for weight and height have underpinned assessment of children’s nutritional status for decades [1, 2], with body mass index (BMI) charts introduced in the 1990s [3]. However, BMI provides no information on the proportions of fat and lean mass [4]. More recently, growth charts for children’s body composition have emerged [5]. Most are based on specific techniques such as skinfolds [6, 7], bioelectrical impedance analysis (BIA) [8–10] and dual-energy X-ray absorptiometry (DXA) [11–14], but multi-component models have also been used [15]. These charts help identify how specific diseases and their treatment impact the body, and may improve the tailoring of treatment regimens and nutritional requirements to individual patient needs [16].

As yet, such reference data primarily address mid-childhood (≥5 y) onwards. Infants and younger children are more difficult to measure, due to poor compliance with most methodologies. Until recently, the main source of information on body composition from birth to 5 years was the reference child [17]. This pioneering study merged multiple data on to US growth centiles, and modeled the development of fat mass, lean mass, and lean tissue components from birth to 10 years, but described only mean values, not the variability.

Body composition reference data for infants have recently been published for several techniques. These include skinfolds [18], DXA [19], air-displacement plethysmography adapted for infants (the Peapod^R^) [20, 21], and a multi-component model [22]. Nevertheless, most of these datasets only address infants < 1 year, though one study covered the first 2 years [22], leaving young children < 5 years poorly addressed. Thus, in younger age groups BMI remains the most commonly used proxy for adiposity, although its limitations for this role are well established [4]. For clinical practice, this is of particular concern because a large proportion of pediatric patients are aged < 5 years. In public health, the first thousand days are now well-established to represent a critical developmental period, during which variability in tissue accretion has major implications for long-term health [23–26]. Both poor infant growth, and rapid weight gain during both infancy and early childhood, have been linked with poorer long-term outcomes [27–29], but data on the underlying body composition trajectories are urgently required, not least because associations of weight gain with later body composition differ between low-/middle-income and high-income settings [30].

We extracted data on total body water (TBW) from multiple studies of infants and children, from which fat-free mass (FFM) and fat mass (FM) can be calculated. We generated new body composition growth charts and standard deviation scores (SDS) for these outcomes and their height-adjusted equivalents, for the age range 6 week to 5 years.

## Methods

Measurements of weight, length or height, and TBW were made in several studies of body composition and energy metabolism conducted by our research group in Cambridge and London, UK between 1987 and 2010. These studies have been described previously [15, 31–36]. Broadly, infants and younger children (<4.5 years) were measured in the early/mid1990s, and the older children from 2001 onwards. Ethical permission for these studies was granted by Cambridge Local Research Ethics Committee, the former MRC Dunn Nutrition Unit, and UCL Great Ormond Street Institute of Child Health. Written informed consent was obtained from parents.

Weight of infants was recorded accurate to 20 g using Seca 727 electronic scales. Length was recorded to 0.2 cm using a Harpenden infantometer. Weight of children was measured on electronic scales accurate to 0.01 kg, and height to 0.1 cm using portable or wall-mounted stadiometers. All data were collected in duplicate, and the average value used. BMI was calculated from weight and height. Data on weight, height, and BMI were converted into standard deviation scores (SDS), using UK 1990 reference data [3, 37], in order to ascertain how representative our sample was of the UK population. BMI was also expressed as SDS relative to WHO reference data [38]. Feeding mode was categorized through parental report as predominantly breast-fed or formula-fed at 6 and 12 weeks. We did not subsequently collect adequate data on breast-feeding status at later ages to allow accurate categorization of feeding mode.

Measurement of TBW requires oral administration of water labeled with ^2^H or ^18^O. After equilibration with the body water pool, the isotopic enrichment of saliva or urine samples can be analyzed to calculate the dilution space (N) using simple dilution principles [39]. There are two approaches to calculating N, known as the plateau method and the back extrapolation method [39]. In children and adults these give similar results, but in infants, where water turnover is more rapid, the plateau method overestimates N, due to dilution of the dose by unlabeled water during equilibration [39]. In our sample, the back extrapolation method was used in all those aged < 2 years, while both methods were used in those aged ≥ 2 years as they gave minimally different values.

Both ^2^H and ^18^O dilution spaces overestimate TBW, due to exchange with non-aqueous exchangeable hydrogen and oxygen. Conventionally, the ^18^O space (N_O_) is converted to TBW by dividing by 1.01, and the ^2^H space (N_D_) by dividing by 1.044 [40]. TBW was therefore calculated as N_O_/1.01 for all infants and children where possible, and as N_D_/1.044 where only the deuterium dilution space was available. We assumed that these adjustments were sufficient to address any inconsistencies in TBW data obtained with the two different isotopes.

FFM was calculated as TBW/hydration, using published age- and sex-specific hydration values [17]. FM was calculated as the difference between FFM and weight. FFM and FM were divided by the square of height, to give Fat-Free Mass index (FFMI) and Fat Mass Index (FMI) in kg/m^2^ units [41]. Seven data points around the age of 2 years were rejected for implausibly high FFMI values.

### Statistics

In order to maximize consistency between these new centiles and those published previously for the age range 5 to 20 years [15], data for children aged 4.2–7.0 years from the latter study (*n* = 81) were included in the analysis.

Sex-specific values by month of age were obtained for body composition outcomes by using the LMS method (LMS Chart Maker; Medical Research Council) [42]. This statistical approach, widely used to construct reference data for traits that incorporate the effects of growth, provides the following 3 outputs: 1) a smoothed median (M or mu) curve, which represents how the outcome varies in relation to age, 2) the CV (S or sigma), which models the scatter of values around the mean and adjusts for any non-uniform dispersion, and 3) the skewness (L or lambda), which corresponds to the age-specific Box-Cox transformation needed to achieve a normal distribution.

TBW and FFM were fitted by using rescaled age, which improves the goodness of fit for monotonic data by fitting the M curve twice. All other outcomes were fitted with transformed age with zero offset, which also improves the fit even for non-monotonic data. Initially, the goodness of fit was assessed by using the Bayesian Information Criterion, with an extra degree of freedom added to the model only if it reduced the deviance by more than log_e_(n) units, where n was the sample size. However, visual inspection of the model-fit was also used, leading to simpler models being selected at the final stage for some outcomes as described in the results. We fitted the data for all ages (6 weeks to 7 years) and derived LMS values and charts for the age range 6 weeks to 5 years, as our earlier work addressed children > 5 years [15]. For all outcomes, we calculated the following SDS cutoffs: −2, −1.67, −1.33, 0, 1.33, 1.67, and 2, which are equivalent to percentiles of 2.3%, 9.2%, 25.2%, 50%, 74.8%, 90.8%, and 97.7% respectively [43]. Finally, we converted previously published body composition reference values from infancy and early childhood [17, 22] into FFM SDS, FM SDS, FFMI SDS and FMI SDS using our own reference data, to compare the datasets.

## Results

**Fig. 1:**
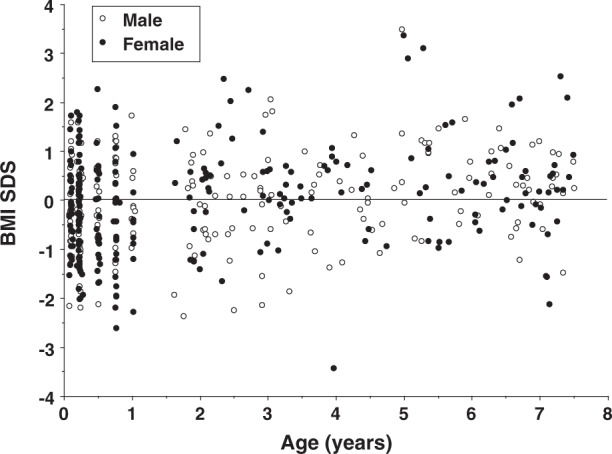
Distribution of body mass index standard deviation score (BMI SDS) against age in the sample. *N* = 199 males, 233 females

Valid body composition data were available for 463 individuals (211 males, 252 females). As shown in Fig. 1, a wide range of BMI SDS was apparent across the age range studied, though the range was narrower for the age range 3 to 5 years. On average, our sample was taller in comparison with UK reference data of the early 1990s (*P* < 0.05 in both sexes), but did not differ in BMI (Table 1). Relative to WHO reference data however, both sexes had greater weight and BMI SDS but were not taller. The numbers predominantly breast-fed and formula-fed were 20 and 29 respectively at 6 weeks, and 45 and 55, respectively at 12 weeks. There were no differences in anthropometry or body composition SDS by infant feeding mode, except at 12 weeks when the breast-fed infants were significantly longer (∆ = 0.44 SDS, 95% CI 0.09, 0.78).

**Table 1:** Anthropometry standard deviation scores relative to the UK 1990 reference in children aged < 5 years

	Males (*n* = 167)				Females (*n* = 200)			
	UK 1990		WHO		UK 1990		WHO	
SDS	∆	95% CI	∆	95% CI	∆	95% CI	∆	95% CI
Weight	0.08	−0.06, 0.22	**0.15**	**0.01**, **0.30**	0.06	−0.07, 0.20	**0.18**	**0.06**, **0.30**
Height	**0.20**	**0.05**, **0.35**	0.07	−0.09, 0.23	**0.24**	**0.09**, **0.39**	0.11	−0.02, 0.25
BMI	−0.10	−0.25, 0.05	0.15	−0.00, 0.30	−0.11	−0.26, 0.03	**0.18**	**0.03**, **0.33**

∆ - difference from zero by paired t-test. Significant results in bold

No significant difference between sexes by two-sample *t* test

Age and BMI SDS were weakly correlated in both sexes (UK reference: *r* = 0.15; WHO reference: *r* = 0.24, both *p* < 0.005). Correlations of FFMI SDS with BMI SDS were 0.49 and 0.54 for males and females respectively, while those of FMI SDS with BMI SDS were 0.49 and 0.56 respectively (all *p* < 0.0001).

Consistent with the narrower BMI SDS range for children aged 3–5 years (Fig. 1), visual inspection of the total body water centiles generated by the best-fit model showed an unexpected dip around the same age range (Supplementary online Fig. 1). The centiles were therefore re-generated with fewer degrees of freedom, reducing sensitivity to age.

**Fig. 2:**
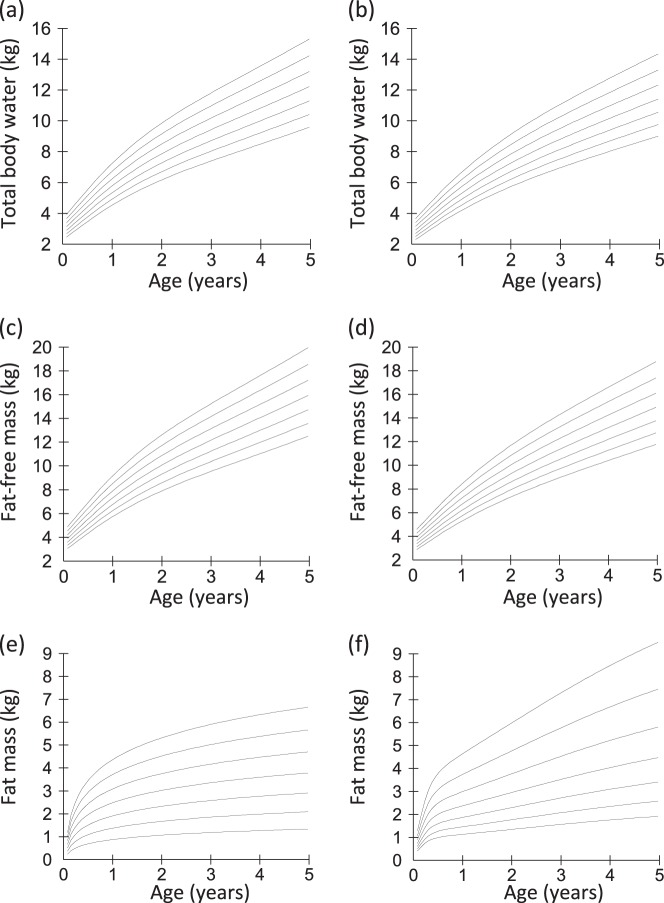
Centiles for total body water (TBW) in (**a**) boys and (**b**) girls, fat-free fat mass (FFM) in (**c**) boys and (**d**) girls, and fat mass (FM) in (**e**) boys and (**f**) girls measured by deuterium dilution. The 3rd, 10th, 25th, 50th, 75th, 90th and 97th centiles are displayed

Based on this approach, LMS centiles for TBW, FFM and FM by sex are shown in Fig. 2. TBW and FFM increased with age in both sexes, but increased faster in males during the first 2 years. In contrast FM centiles were similar between the sexes, with a dramatic fall in fat accretion after the first 3 months. The median boy and girl had accumulated ~2.5 kg fat by 1 year, but only another ~1 kg by 4.5 years. Supplementary online Table 1 provides LMS values by 1-month intervals for each sex.

**Fig. 3:**
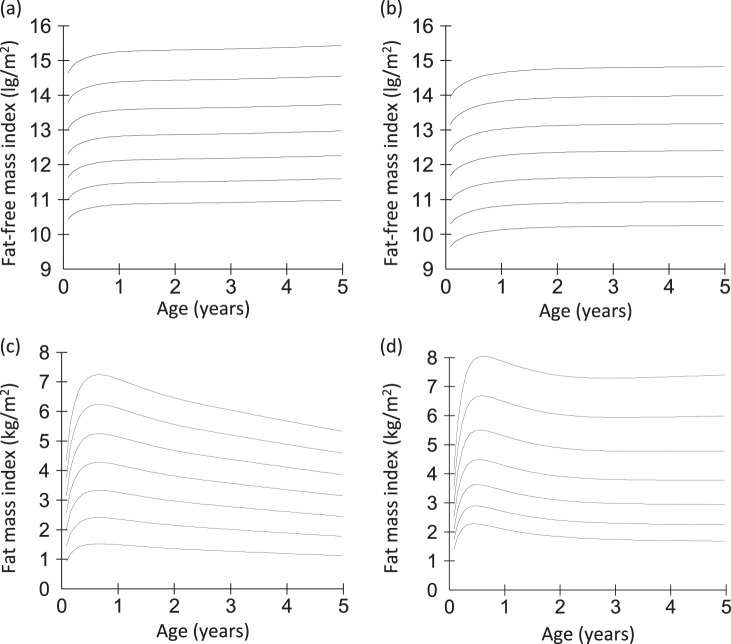
Centiles for (**a**) fat-free mass index (FFMI) in boys, (**b**) fat-free mass index (FFMI in girls, (**c**) fat mass index (FMI) in boys and (**d**) fat mass index (FMI) in girls. The 3rd, 10th, 25th, 50th, 75th, 90th and 97th centiles are displayed

LMS centiles for FFMI and FMI by sex are shown in Fig. 3. Male FFMI plateaued around 1 year, indicating that FFM accretion outstrips length growth during early life, with this pattern subsequently fading. A weaker version of this pattern was evident in females, so that FFM accretion was broadly proportional to height from 1 year onwards. In both sexes, FMI rose up to ~6 months and then declined, first rapidly then more slowly, so that the slope was almost flat by 2 years in both sexes. This indicates that fat was gained in proportion with height during early childhood.

**Fig. 4:**
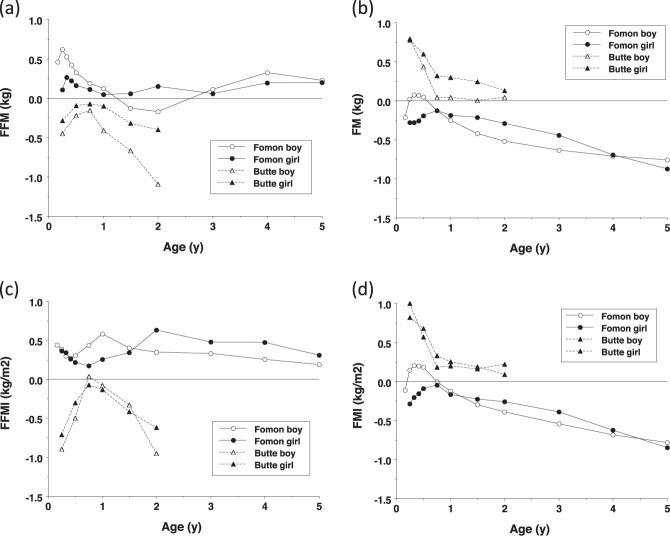
Published data of Fomon and colleagues [17] and Butte and colleagues [22] converted to z-scores using our new reference data, to evaluate differences between studies in (**a**) fat-free mass (FFM), (**b**) fat mass (FM), (**c**) fat-free mass index (FFMI) and (**d**) fat mass index (FMI)

Figure 4 plots the reference child of Fomon and colleagues [17] and the data of Butte and colleagues 2000 [22] as SDS relative to our data. The Fomon data showed broadly similar age trends for FFM, though ~0.5 SDS greater when expressed as FFMI, and similar levels of fatness in infancy, but progressively lower values from 6–9 months onwards. The Butte data showed lower FFM and FFMI values in early infancy and after 1 year, but similar values in the second 6 months. Fatness was substantially higher in early infancy, but was similar to our values from 9 months.

Consistency between the new TBW centiles and those published previously for children aged > 5 years was assessed by graphic analysis (Supplementary online Fig. 2). Even though the two charts included 81 children in common across the age range at which they intersect, the new centiles were lower in the younger children by around ~0.33 SDS in the lower centiles and ~0.66 SDS in the upper centiles.

## Discussion

We present the first body composition reference data using a two-component model over the combined period of infancy and early childhood. This enables trajectories of FM and FFM accretion to be described over the crucial first thousand days of life, a period when growth is sensitive to diverse ecological stimuli and stresses, and has implications for long-term health. Beyond the overall patterns of development, our charts also highlight subtle sex differences in FFM and FM accretion. In early infancy, though weight gain is slowing, males gain FFM faster than females, who show slightly faster FM accretion. From 3 years, females gain both tissues at consistent rates, whereas FM accretion declines in males while FFM accretion increases.

Previously, evaluations of early growth patterns were restricted to anthropometric outcomes, most commonly weight, height and body mass index [3, 37], but also skinfold thicknesses [6]. These approaches can describe increases in components of size, and regional adiposity, but provide no direct information on body composition in terms of differentiating FM and FFM. While skinfolds evaluate regional subcutaneous adiposity at specific regional depots, they relate poorly to whole body fat in infancy [44]. Most importantly, none of these approaches provides useful information about FFM, the primary functional component of body mass.

These reference data broadly precede the period during which the prevalence of obesity has increased in younger children. Even in the 1990s, the children we studied were on average heavier and taller than the British reference data, but importantly, there was no difference in BMI. Relative to WHO data, both our male and female sample average ~0.2 z-scores for BMI, still a relatively small difference.

A potential limitation of our charts, however, is that the older children were measured ~10 years more recently than the infants. On this basis, the charts might potentially express as age-associated increases in fat or lean mass increments that are actually due to secular trends in nutritional status. The widening difference between adiposity z-scores with age, when comparing our sample to that of Fomon, might seem to support that interpretation. However, life-course exposure to obesogenic factors also inherently increases with age, hence the specific contribution of secular trends may be modest.

Nevertheless, despite incorporating 81 data-points from our previously published reference data for older children, with the aim of making the centile charts overlap successfully, a poor join was apparent for TBW centiles above the median, which would propagate to all other body composition outcomes. We suggest that this is partly because older children have had longer exposure to obesogenic factors than younger children; and partly because the older children were measured more recently and have consistently experienced an overall more obesogenic environment. Since greater FM tends to be accompanied by higher FFM (and hence TBW), both of these scenarios contribute to greater distances between the upper centiles in the more recently-measured older children, compared to the younger children. Overall, this indicates that individuals and groups cannot be monitored across the two charts, rather each should be used separately. Further work may be conducted to merge the two datasets, however the different historical periods of recruitment cannot be resolved.

More generally, the comparisons with previously published data indicate substantial population variability in body composition in early life. Our children’s data appear to reflect a more obesogenic setting compared to Fomon’s reference child [17], reflected in progressively higher FM in the UK with increasing age. The Butte data [22] show larger differences in infancy in both tissues, which are harder to interpret. Possible contributing factors may be varying contributions of breast- vs formula-feeding, or differences in measurement methods (eg we used the back extrapolation method in infants, whereas Butte at el used both this and the plateau method). Since these contrasts were present both in early infancy for FM, and in late infancy for FFM, we suspect that population differences in tissue accretion patterns are likely to be one contributing factor. On this basis, our data should not be considered definitive, rather they give a broad indication of early tissue accretion patterns.

Nevertheless, these reference data should be of value to clinicians, given that a large proportion of pediatric hospital patients are aged < 5 years. Infants and young children, especially when sick, may struggle to comply with most measurement protocols. Currently available techniques include BIA, DXA and air-displacement plethysmography (ADP) [45, 46]. Each has some limitations: ADP and DXA require access to expensive non-portable instrumentation, and ADP is currently available only for infants < 6 months of age and > 2 years of age, while DXA involves exposure to very low levels of radiation. BIA is a more widely available technique and is portable as well as cheap and quick to use, however not all infants and young children stay sufficiently still to satisfy the protocol. Moreover, raw BIA data are converted to final body composition values using prediction equations that should ideally be generated in the study population, or obtained from studies on similar populations. In contrast, isotope techniques are relatively easy to perform on all human subjects. The main limiting factors for this approach are the cost, and the delay in obtaining results. In addition, where patients may have dehydration, or over-hydration from edema, measurements of TBW may remain difficult to interpret. Rapid clinical services could be developed using simpler analytical techniques, such as Fourier-Transform Infrared Spectropotometry.

Our reference data may also be of value in the context of public health. There is increasing realization that infants and children differ amongst each other in their relative accretion of weight and length, however the conversion of data to BMI provides data on nutritional status rather than body composition. Recent studies have identified peak BMI velocity in infancy as an important predictor of subsequent obesity risk [44], nevertheless it remains difficult to know whether this implicates the accretion of fat tissue during early life, or simply a faster rate of growth.

## Supplementary information

Supplementary material

Reference Data

## References

[1] Tanner JM (1952). The assessment of growth and development in children. Arch Dis Child.

[2] Tanner JM, Whitehouse RH (1976). Clinical longitudinal standards for height, weight, height velocity, weight velocity, and stages of puberty. Arch Dis Child.

[3] Cole TJ, Freeman JV, Preece MA (1995). Body mass index reference curves for the UK, 1990. Arch Dis Child.

[4] Wells JC (2000). A Hattori chart analysis of body mass index in infants and children. Int J Obes Relat Metab Disord.

[5] Wells JC (2014). Toward body composition reference data for infants, children, and adolescents. Adv Nutr.

[6] Tanner JM, Whitehouse RH (1975). Revised standards for triceps and subscapular skinfolds in British children. Arch Dis Child.

[7] Moreno LA, Mesana MI, Gonzalez-Gross M, Gil CM, Ortega FB, Fleta J (2007). Body fat distribution reference standards in Spanish adolescents: the AVENA Study. Int J Obes (Lond).

[8] Chumlea WC, Guo SS, Kuczmarski RJ, Flegal KM, Johnson CL, Heymsfield SB (2002). Body composition estimates from NHANES III bioelectrical impedance data. Int J Obes Relat Metab Disord.

[9] Kurtoglu S, Mazicioglu MM, Ozturk A, Hatipoglu N, Cicek B, Ustunbas HB (2010). Body fat reference curves for healthy Turkish children and adolescents. Eur J Pediatr.

[10] Nakao T, Komiya S (2003). Reference norms for a fat-free mass index and fat mass index in the Japanese child population. J Physiol Anthropol Appl Human Sci.

[11] Weber DR, Moore RH, Leonard MB, Zemel BS (2013). Fat and lean BMI reference curves in children and adolescents and their utility in identifying excess adiposity compared with BMI and percentage body fat. Am J Clin Nutr.

[12] van der Sluis IM, de Ridder MA, Boot AM, Krenning EP, de Muinck Keizer-Schrama SM (2002). Reference data for bone density and body composition measured with dual energy x ray absorptiometry in white children and young adults. Arch Dis Child.

[13] Alwis G, Rosengren B, Stenevi-Lundgren S, Duppe H, Sernbo I, Karlsson MK (2010). Normative dual energy X-ray absorptiometry data in Swedish children and adolescents. Acta Paediatr.

[14] Khadilkar AV, Sanwalka NJ, Chiplonkar SA, Khadilkar VV, Pandit D (2013). Body fat reference percentiles on healthy affluent Indian children and adolescents to screen for adiposity. Int J Obes (Lond).

[15] Wells JC, Williams JE, Chomtho S, Darch T, Grijalva-Eternod C, Kennedy K (2012). Body-composition reference data for simple and reference techniques and a 4-component model: a new UK reference child. Am J Clin Nutr.

[16] Wells JC, Fewtrell MS (2008). Is body composition important for paediatricians?. Arch Dis Child.

[17] Fomon SJ, Haschke F, Ziegler EE, Nelson SE (1982). Body composition of reference children from birth to age 10 years. Am J Clin Nutr.

[18] de Onis M, Onyango AW, Borghi E, Siyam A, Nishida C, Siekmann J (2007). Development of a WHO growth reference for school-aged children and adolescents. Bull World Health Organ.

[19] Rigo J, Nyamugabo K, Picaud JC, Gerard P, Pieltain C, De CM (1998). Reference values of body composition obtained by dual energy X-ray absorptiometry in preterm and term neonates. J Pediatr Gastroenterol Nutr.

[20] Fields DA, Gilchrist JM, Catalano PM, Gianni ML, Roggero PM, Mosca F (2011). Longitudinal body composition data in exclusively breast-fed infants: a multicenter study. Obes (Silver Spring).

[21] Andersen GS, Girma T, Wells JC, Kaestel P, Leventi M, Hother AL (2013). Body composition from birth to 6 mo of age in Ethiopian infants: reference data obtained by air-displacement plethysmography. Am J Clin Nutr.

[22] Butte NF, Hopkinson JM, Wong WW, Smith EO, Ellis KJ (2000). Body composition during the first 2 years of life: an updated reference. Pediatr Res.

[23] Victora CG, Adair L, Fall C, Hallal PC, Martorell R, Richter L (2008). Maternal and child undernutrition: consequences for adult health and human capital. Lancet.

[24] Adair LS, Fall CH, Osmond C, Stein AD, Martorell R, Ramirez-Zea M (2013). Associations of linear growth and relative weight gain during early life with adult health and human capital in countries of low and middle income: findings from five birth cohort studies. Lancet.

[25] Yajnik CS, Fall CH, Coyaji KJ, Hirve SS, Rao S, Barker DJ (2003). Neonatal anthropometry: the thin-fat Indian baby. The Pune Maternal Nutrition Study. Int J Obes Relat Metab Disord.

[26] Wells JC The metabolic ghetto: an evolutionary perspective on nutrition, power relations and chronic disease. Cambridge: Cambridge University Press, 2016.

[27] Barker DJ, Winter PD, Osmond C, Margetts B, Simmonds SJ (1989). Weight in infancy and death from ischaemic heart disease. Lancet.

[28] Eriksson JG, Forsen T, Tuomilehto J, Winter PD, Osmond C, Barker DJ (1999). Catch-up growth in childhood and death from coronary heart disease: longitudinal study. BMJ.

[29] Taveras EM, Rifas-Shiman SL, Belfort MB, Kleinman KP, Oken E, Gillman MW (2009). Weight status in the first 6 months of life and obesity at 3 years of age. Pediatrics.

[30] Wells JC, Chomtho S, Fewtrell MS (2007). Programming of body composition by early growth and nutrition. Proc Nutr Soc.

[31] Davies PS, Ewing G, Lucas A (1989). Energy expenditure in early infancy. Br J Nutr.

[32] Wells JC, Ritz P, Davies PS, Coward WA (1998). Factors affecting the 2H to 18O dilution space ratio in infants. Pediatr Res.

[33] Wells JC, Ritz P (2001). Physical activity at 9-12 months and fatness at 2 years of age. Am J Hum Biol.

[34] Wells JC, Stanley M, Laidlaw AS, Day JM, Davies PS (1996). The relationship between components of infant energy expenditure and childhood body fatness. Int J Obes Relat Metab Disord.

[35] Davies PS, Coward WA, Gregory J, White A, Mills A (1994). Total energy expenditure and energy intake in the pre-school child: a comparison. Br J Nutr.

[36] Wells JC, Fuller NJ, Wright A, Fewtrell MS, Cole TJ (2003). Evaluation of air-displacement plethysmography in children aged 5-7 years using a three-component model of body composition. Br J Nutr.

[37] Freeman JV, Cole TJ, Chinn S, Jones PR, White EM, Preece MA (1995). Cross sectional stature and weight reference curves for the UK, 1990. Arch Dis Child.

[38] W. H. O. Multicentre Growth Reference Study Group. (2006). WHO Child Growth Standards based on length/height, weight and age. Acta Paediatr Suppl.

[39] Davies PS, Wells JC (1994). Calculation of total body water in infancy. Eur J Clin Nutr.

[40] Racette SB, Schoeller DA, Luke AH, Shay K, Hnilicka J, Kushner RF (1994). Relative dilution spaces of 2H- and 18O-labeled water in humans. Am J Physiol.

[41] VanItallie TB, Yang MU, Heymsfield SB, Funk RC, Boileau RA (1990). Height-normalized indices of the body’s fat-free mass and fat mass: potentially useful indicators of nutritional status. Am J Clin Nutr.

[42] Cole TJ, Freeman JV, Preece MA (1998). British 1990 growth reference centiles for weight, height, body mass index and head circumference fitted by maximum penalized likelihood. Stat Med.

[43] Cole TJ (1994). Do growth chart centiles need a face lift?. BMJ.

[44] Davies PS, Lucas A (1990). The prediction of total body fatness in early infancy. Early Hum Dev.

[45] Wells JC, Fewtrell MS (2006). Measuring body composition. Arch Dis Child.

[46] Ellis KJ, Yao M, Shypailo RJ, Urlando A, Wong WW, Heird WC (2007). Body-composition assessment in infancy: air-displacement plethysmography compared with a reference 4-compartment model. Am J Clin Nutr.

