# Supplementary material for: Body composition reference charts for UK infants and children aged 6 weeks to 5 years based on measurement of total body water by isotope dilution
Source: Eur J Clin Nutr. 2019 Feb 26;74(1):141–8. doi: 10.1038/s41430-019-0409-x (PMC6949189; doi:10.1038/s41430-019-0409-x)
Supplement: Supplementary file 1 — Supplementary material [file 41430_2019_409_MOESM1_ESM.pdf]

Supplementary online material

Body composition reference charts for UK infants and children aged 6 weeks to 5 years based on measurement of total body water by isotope dilution; Wells et al.

Supplementary online Figure 1. Centiles generated by (a) the best fit according to the Bayesian Information Criterion, and (b) a simpler model with fewer degrees of freedom in order to correct for sampling bias over the age range 3 to 5 years

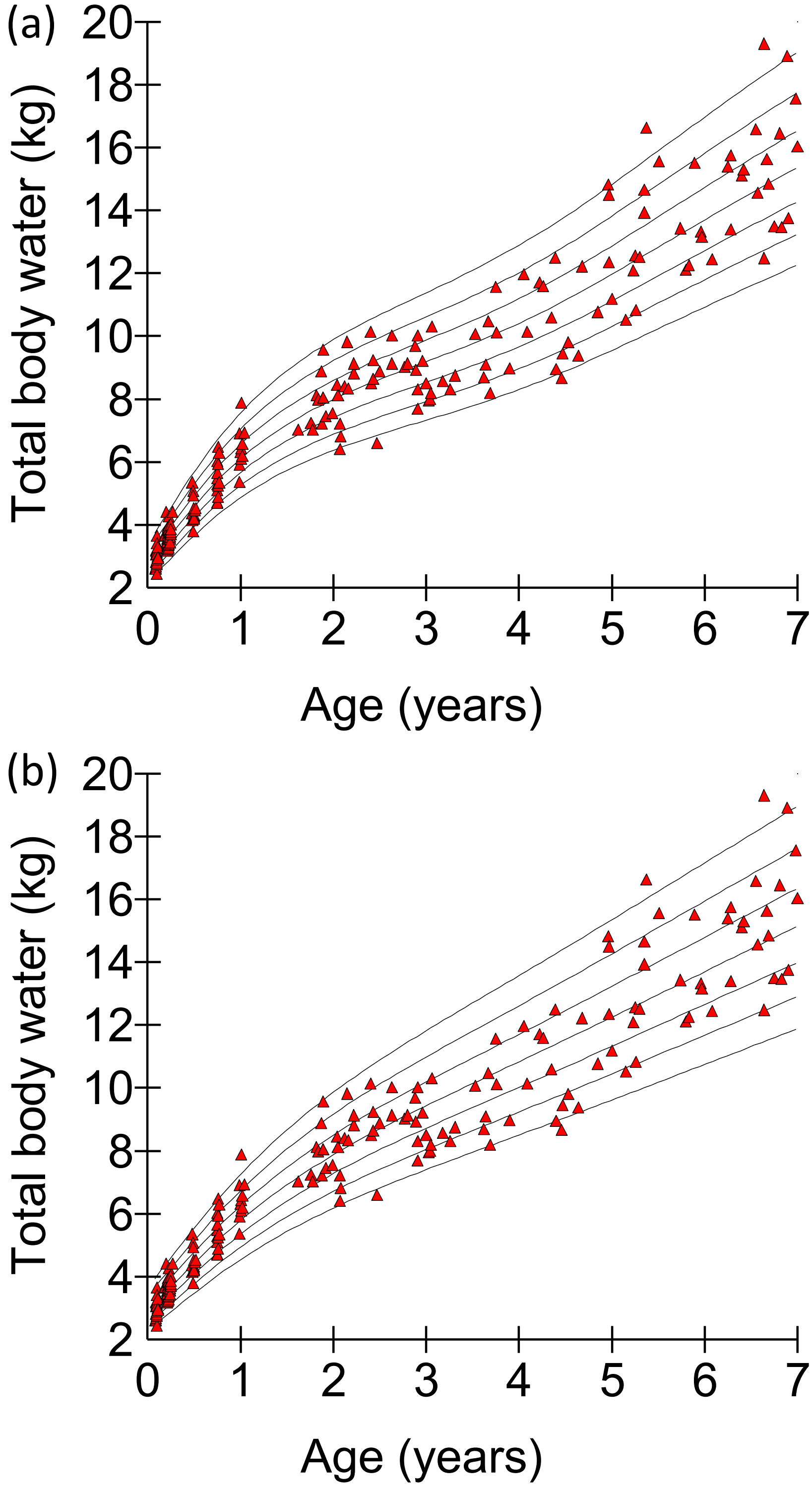

Supplementary online Figure 2. Total body water reference curves for new data <5 years and published data >5 years Wells et al., Am J Clin Nutr 2012; 96(6):1316-26

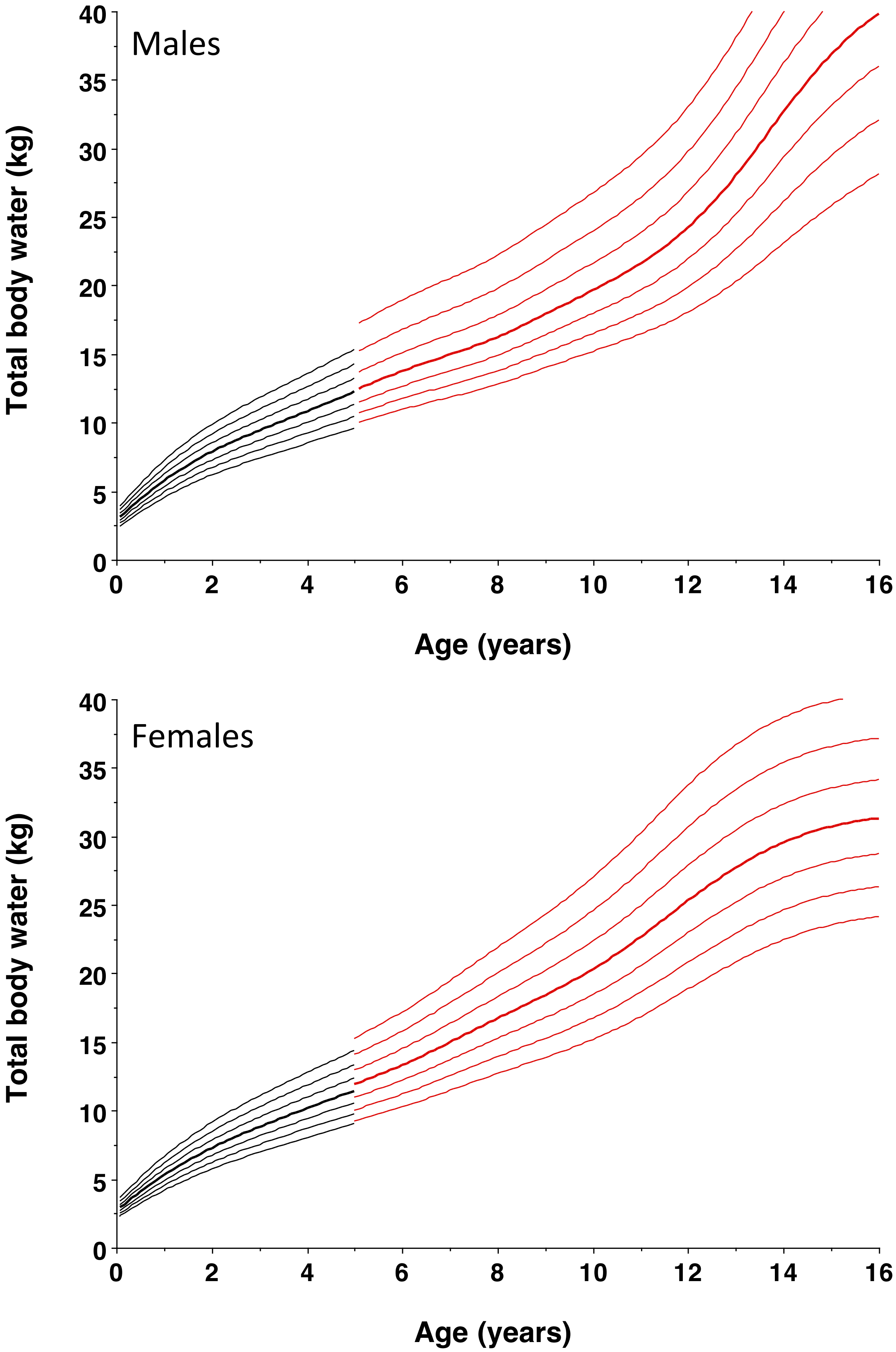

Supplementary online Table 1.
Numbers of infants and children analysed by age for each sex

	Males (n)	Females (n)	Sexes combined (n)
1.5 months	20	29	49
3 months	41	51	92
6 months	13	24	37
9 months	13	25	38
12 months	10	8	18
1.5 – 1.99 years	12	10	22
2 - 2.99 years	25	23	48
3 - 3.99 years	16	20	36
4 - 4.99 years	16	10	26
5 - 5.99 years	16	15	31
6 - 6.99 years	18	21	39
7 - 7.50 years	11	16	27
Total	211	252	463

All measurements represented new data points, except that those measured at 1.5 months were re-measured at 3, 6 and 9 months. The numbers of these repeat measurements were 50 at 3 months (54% of the sample), 37 at 6 months (100% of the sample), and 22 at 9 months (58% of the sample).
